# What is a Fresh Scent in Perfumery? Perceptual Freshness is Correlated with Substantivity

**DOI:** 10.3390/s130100463

**Published:** 2012-12-28

**Authors:** Manuel Zarzo

**Affiliations:** Department of Applied Statistics, Operations Research and Quality, Universidad Politécnica de Valencia, Cmno. Vera s/n, 46022 Valencia, Spain; E-Mail: mazarcas@eio.upv.es; Tel.: +34-963-877-490; Fax: +34-963-877-499

**Keywords:** fragrance, tenacity, odor descriptor, perceptual dimension, olfactory map

## Abstract

Perfumes are manufactured by mixing odorous materials with different volatilities. The parameter that measures the lasting property of a material when applied on the skin is called substantivity or tenacity. It is well known by perfumers that citrus and green notes are perceived as fresh and they tend to evaporate quickly, while odors most dissimilar to ‘fresh’ (e.g., oriental, powdery, erogenic and animalic scents) are tenacious. However, studies aimed at quantifying the relationship between fresh odor quality and substantivity have not received much attention. In this work, perceptual olfactory ratings on a fresh scale, estimated in a previous study, were compared with substantivity parameters and antierogenic ratings from the literature. It was found that the correlation between fresh odor character and odorant substantivity is quite strong (*r* = −0.85). ‘Fresh’ is sometimes interpreted in perfumery as ‘cool’ and the opposite of ‘warm’. This association suggests that odor freshness might be somehow related to temperature. Assuming that odor perception space was shaped throughout evolution in temperate climates, results reported here are consistent with the hypothesis that ‘fresh’ evokes scents typically encountered in the cool season, while ‘warm’ would be evoked by odors found in nature during summer. This hypothesis is rather simplistic but it may provide a new insight to better understand the perceptual space of scents.

## 1. Introduction

*Fresh* is an odor character descriptor commonly used in perfumery. Actually, it is the one most frequently encountered in a semantic olfactory database of 119 perfume materials reported by Thiboud [1]. In a recent study of this database, it was found that *warm*, *oriental* and *powdery* were the descriptors most dissimilar to *fresh* (see Figure 6 of [2]). Consistent with this result, *fresh* and *oriental* are regarded as opposite families of perfumes in the Fragrance Wheel proposed by Edwards [3]. The *fresh* category comprises *citrus*, *green*, *water*, and *fruity* subfamilies.

The Odor Effects Diagram is an olfactory representation of perfumery notes based on two basic polarities: (i) erogenous *vs.* antierogenous (refreshing) and (ii) narcotic *vs.* stimulating [4]. *Citrus*, *green*, *watery*, and *aldehydic* are regarded as refreshing scents, while *erogenous*, *animal*, *musk*, *vanilla* and *powdery* appear at the opposite pole (see Figure 5 of [2]). *Fruity* is located in this diagram between *floral* and *fresh*, and the same criterion was considered by Edwards [3].

Perfume is a complex mixture of odorants with different volatilities. The parameter that measures the lasting property of a material when applied on the skin is called substantivity or tenacity. It is well known by perfumers that olfactory notes perceived as fresh tend to evaporate quickly, while the opposite applies to those most dissimilar to *fresh*. Actually, *fresh* and *green* are attributes commonly encountered in the description of top notes (*i.e.*, the ones that are perceived firstly when smelling a fragrance) [2]. *Light* refers to scents with high volatility, while *heavy*, *rich* or *tenacious* is applied to materials with high substantivity. Light fragrances are those perceived as non-sweet with a predominant fresh note that is often associated with citrus, greens or aldehydes [5]. Conversely, the least volatile ingredients such as mosses and animal scents dominate in heavy perfumes [1]. Vapor pressure is the basic factor that determines the volatility of a specific compound [6], but the vapor composition in equilibrium with the liquid is difficult to predict in mixtures due to the complex molecular interactions that occur [7–10].

Sensory ratings on a scale of freshness are difficult to obtain because the fresh dimension of olfactory perception is not well understood yet. Probably for this reason, psychophysical studies aimed at quantifying the relationship between this odor quality and tenacity have not received much attention yet. Several sensory maps of scents reported in the literature are investigated in the present work attempting to further understand the psychological aspects involved in the perception of refreshing odor character. The main target of the present work is to study the correlation between this odor quality and odorant substantivity. This relationship is well established in perfumery, but only at descriptive level.

## 2. Methods

In order to characterize the connection between perceived fresh odor character and substantivity, three stages have been carried out: (i) to estimate the freshness of odorant materials and descriptors on a numeric scale, (ii) to obtain substantivity values from the literature, and (iii) to study the correlation between them by means of simple linear regression. Each one of these stages is detailed below in a different section. In the present work, ‘fresh’ and ‘refreshing’ are used as synonyms. The different connotations of both terms are discussed in Section 4.4.

### 2.1. Classification of Odor Descriptors on a Scale of Perceptual Freshness

Hedonic tones of odor descriptors are available from the literature, and they can be used to interpret underlying dimensions of odor profile databases [11]. Similarly, it is possible to assign a *refreshing* tone to odor descriptors as described ahead. Boelens and Haring [12] asked a panel of six perfumers to smell 309 aroma chemicals and to rate on a 0–9 scale the odor similarity to 30 reference materials. Each one was selected as a standard for a certain odor character descriptor. The resulting sensory dataset, which will be referred to hereafter as B-H database, was analyzed in previous studies using principal components analysis (PCA) [2,11]. The first principal component (PC1) is the linear combination of variables (reference materials) that explains the maximum amount of data variability (17.5% in this case). The contributions of variables in the formation of a given PC are called loadings, being p_1_ and p_2_ the loadings corresponding to PC1 and PC2, respectively. PC1 was interpreted as a dimension of freshness because *fresh* was the descriptor with highest p_1_ loading. The p_1_ values can be regarded as (i) indirect assessments of the 30 reference materials on a scale of odor freshness, and (ii) *refreshing* tones of the odor descriptors associated to the reference materials.

Thiboud [1] reproduces a two-dimensional projection of a similarity matrix developed from interviews with consumers in England, USA, Spain, Brazil, and Japan relating to defined perfume bases and to verbal descriptions. The horizontal axis of this fragrance mapping discriminates masculine *vs.* feminine scents, and *fresh* is one of the descriptors that determine the vertical axis. The same interpretation was proposed for PC2 and PC1 of the B-H database [2]. Thus, a scatterplot of p_2_*vs.* p_1_, which is usually referred to as loading plot, was superimposed with Thiboud's sensory map, after being properly scaled and rotated in order to achieve the best matching between both odor representations.

Chastrette *et al.*[13] analyzed semantic odor profiles of 628 pure odorous substances commonly encountered in perfumery. The database was obtained by Firmenich SA from a team of seven perfumers who assessed each compound and assigned two to four notes chosen among 32 possible descriptors. The three most frequent ones were considered as the odor profile. A transformed matrix derived from this database was analyzed with PCA, and the PC1/PC2 loading plot was proposed as a sensory map of scents. I obtained the projections over the first factorial axis from this plot (Figure 1 of [13]) and compared these projections with p_1_ loadings of equivalent descriptors in the B-H database.

Different sensory maps of scents have been developed by chemical companies that supply perfume raw materials. One of them is the Rosace of Firmenich [14], which is comprised by 13 categories of scents properly arranged according to the opinion of professional perfumers. *Citrus* and *balsamic* are located at opposite positions, which suggests that this direction can be interpreted as a dimension of freshness. I obtained the orthogonal projections of all odor classes over this direction. Next, the correlation between these projections and p_1_ loadings from the B-H database was studied.

Another olfactory representation is the Field of Odors, which displays in a semicircle different descriptors and odor categories [15]. It was derived from an odor profile database of perfume materials. Attempting to study if freshness is a salient dimension of this odor map, I obtained the polar coordinates for the different odor classes (*i.e.*, angular position in the semicircle and distance to the center). The angular coordinates were compared with p_1_ loadings of equivalent attributes in the B-H database.

### 2.2. Fresh Odor Quality and Antierogenic Character of Odorant Materials

Thiboud [1] compiled semantic odor profiles for 44 natural odorants and 75 synthetic chemicals. Each material was labeled with three or four main odor descriptors and with a set of ancillary attributes ranging from 0 to 14 (average = 6.6). This database was analyzed in a previous study [2] and it was found that freshness was an underlying dimension. Based on a few representative descriptors of this dimension, I obtained estimated scores on a fresh scale for the 44 natural odorants as described below.

Jellinek [4] conducted a sensory study attempting to classify perfume materials as erotic or not erotic. Three experienced perfumers assessed the erogenic character of 61 essential oils, 34 extracts and 105 aroma chemicals. Each material was added to four perfume compositions at a level that was just sufficient to create a clearly noticeable difference, and the panel had to decide whether the added material increased or decreased the erotic character of the perfume. From these sensory ratings, an antierogenic index (AI) was derived ranging from −100 to 100. The maximum value 100 indicates that the material was regarded by the panel as clearly antierogenic, while −100 corresponds to a noticeably erogenic scent. Jellinek [4] considered that *antierogenic* could also be interpreted as *refreshing* in odor description. To study this issue, I checked the correlation between p_1_ and AI of reference materials in the B-H database.

### 2.3. Substantivity Parameters of Perfume Materials

After conducting an extensive literature review, I found four substantivity parameters of perfume materials as described next. These parameters were not obtained in the same way and may not be accurate, but they are correlated and can be compared with perceptual freshness.

Perfumes are described according to their top, middle and base notes, which are also called head, heart and bottom notes, respectively. The top note is the first odor perceived when smelling a fragrance product, and it usually consists of the most volatile portion of the composition [1]. Middle notes, which represent the main body of a blend, are perceived after the top notes fade away [5]. Base notes are basically determined by fixative materials with a very low volatility and great substantivity that yield the characteristic lasting note of any fragrance. The three stages of evaporation are often illustrated with a pyramid [16]. The H&R Fragrance Guide [17] contains the semantic odor description of 820 commercial perfumes (367 men's and 453 women's). I counted the total number of times that a given attribute was applied to describe top (N_top_), middle (N_mid_) and base notes (N_base_). A substantivity index was calculated according to Equation (1), which takes the value 0, 50 and 100 for attributes that are only applied to describe top, middle and base notes, respectively. Thus, it provides an estimation of the substantivity associated to a given descriptor on a 0-100 scale:

(1) $$\text{Substantivity index}=\frac{50\cdot {\text{N}}_{\text{mid}}+100\cdot {\text{N}}_{\text{base}}}{{\text{N}}_{\text{top}}+{\text{N}}_{\text{mid}}+{\text{N}}_{\text{base}}}$$

Poucher [18] obtained the duration of evaporation by olfaction for 332 odorous materials. A certain quantity of each odorant was placed on a paper strip, and he determined the time that the smelling strip retained the typical odor note. Based on the results, a coefficient of substantivity (CS) from 1 to 100 was assigned to each material.

Another substantivity parameter was reported by Appell [7], who calculated the volatility of 81 essential oils by obtaining the amount of oil evaporated in various time intervals. The quantity (mg) evaporated in one hour from one gram of oil was called evaporation index.

The website of *The Good Scents Company* (www.thegoodscentscompany.com) provides useful information about suppliers, safety, organoleptic properties, and physical parameters of aroma chemicals and natural perfume materials. Substantivity values, which will be referred as S_GS_, are available for most odorants. This parameter is measured in hours and ranges from 1 to 400 h. The maximum S_GS_ in fact indicates a censored value higher than 400 (*i.e.*, ≥16.7 days). Actually, certain materials can smell for months [8].

### 2.4. Relationship between Fresh Odor Character and Substantivity

The p_1_ loadings derived from the B-H database can be regarded as assessments of the 30 reference materials on a perceptual scale of odor freshness. These values can also be interpreted as *refreshing* tones of the odor descriptors associated to reference materials (*i.e.*, the degree of perceptual similarity or dissimilarity of a given odor quality with respect to the fresh odor character). The correlation between p_1_ loadings and the substantivity index of equivalent descriptors was studied by means of linear regression. The evaporation index and S_GS_ are available for most reference materials in the B-H database. Thus, the correlation between p_1_, EI and S_GS_ was also checked.

CS values are available for the 44 odorants used by Thiboud [1]. I studied the correlation of CS with the scores of freshness derived from odor profiles, as well as with AI and other substantivity parameters. Additional materials commonly used in perfumery were also taken into consideration.

## 3. Results

The comparison of *refreshing* tones derived from odor profiles and sensory maps yields consistent results, as indicated in the next section. This issue is of interest to further understand the psychological issues involved in the perception of fresh odor character. Section 3.2 describes the correlation between the fresh odor character and substantivity.

### 3.1. Freshness as an Underlying Dimension in Odor Maps

**Figure 1.**
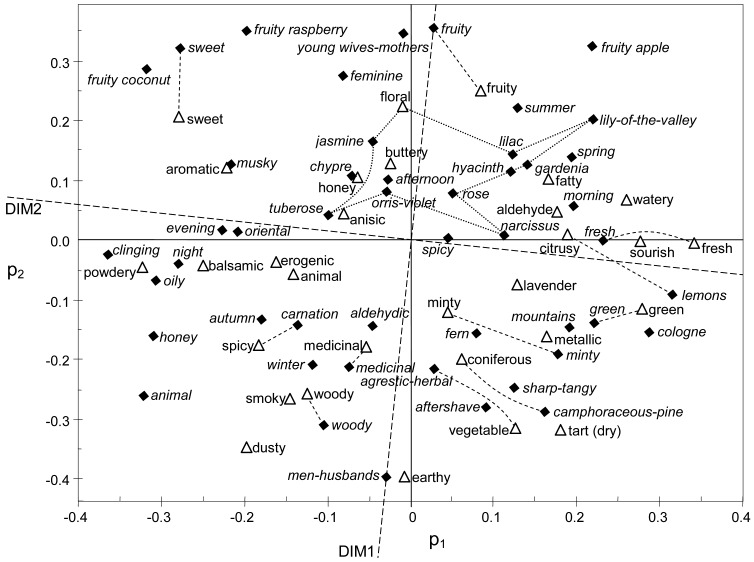
Loading plot (p_2_*vs.* p_1_) of the database obtained by Boelens & Haring [12] (white triangles). Data were mean-centered and scaled to unit variance prior to the PCA. Thiboud's fragrance map [1] is superimposed (filled diamonds; labels in italics) after being properly scaled and rotated (DIM1 and DIM2 stand for dimension 1 and 2, respectively, in the original publication). Dotted lines group the floral descriptors. Equivalent or related odor attributes located close to each other are joined with dashed lines.

Figure 1 shows the PC1/PC2 loading plot of the B-H database, superimposed with a rotated fragrance map [1]. Although the position of comparable descriptors is not exactly coincident, both sensory maps are strikingly similar. An odor map based on equivalent dimensions has also been reported [19]. Certain disparity exists in Figure 1 for some attributes (e.g., *spicy*, *aldehydic*, *honey*, or *animal*) probably due to the lack of consensus in their interpretation. For example, eugenol and cinnamon are frequently chosen by perfumers as a reference for *spicy*[20], but the latter smells sweeter. Decanal was the reference for *aldehydic* in the B-H database. It smells antierogenic, but most aldehydes are perceived as erogenic [4], which would explain the disparity of *aldehydic*. Honey scents are often described in perfumery as sweet-medicinal, and Abe *et al.*[21] found a similarity between *honey* and *animal*, which would clarify the discrepancy of *honey* in Figure 1.

Regarding the vertical axis of Figure 1, *men-husbands* appears at the bottom and *feminine* is found at the opposite side. This result further supports the hypothesis of a dimension influenced by psychological and cultural aspects that discriminates feminine *vs.* masculine cosmetic odors, as discussed elsewhere [2,19].

**Figure 2.**
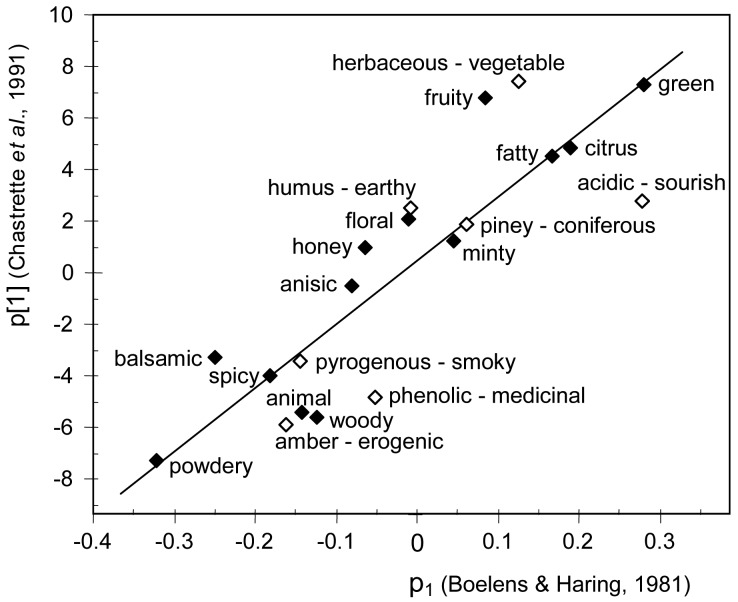
Plot of fitted regression analysis of *p_[1]_* (projections on the first factorial axis) from the Firmenich database analyzed by Chastrette *et al.*[13]*vs.* p_1_ of the B-H database. Empty diamonds correspond to pairs of similar descriptors (e.g., ‘acidic—sourish’ are assumed to be equivalent attributes in the Firmenich and the B-H databases, respectively).

Another sensory map of scents was obtained by Chastrette *et al.*[13]. Most descriptors in this odor map have a direct correspondence with attributes in the B-H database. *Herbaceous* was paired with *vegetable* because the reference for the latter smells like herbs [2]. *Medicinal* was matched with *phenolic* because a significant similarity between both descriptors was found in a previous study [22]. *Amber* was paired with *erogenic* because a mixture of ambergris and costus oil was the reference material for *erogenic* in the B-H database. Figure 2 compares the projection of olfactory notes over the first factorial axis obtained by Chastrette *et al.*[13] with p_1_ loadings of equivalent descriptors in the B-H database. The correlation is statistically significant (*r* = 0.86, *p* < 10^−4^), which reveals that freshness is the most salient dimension.

**Figure 3.**
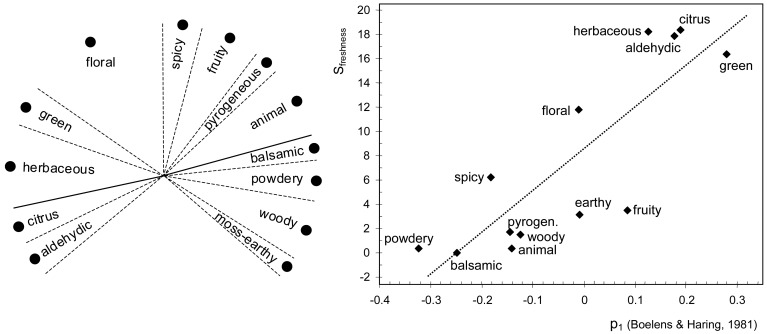
(**left**) Olfactory representation adapted from the Rosace of Firmenich [14]. (**right**) Projections of dots over the solid line (S_freshness_) are compared with p_1_ loadings of equivalent attributes in the B-H database.

Figure 3 shows the 13 odor classes contained in the Rosace of Firmenich [14]. *Citrus* and *aldehydic* are located in opposite positions with respect to *balsamic* and *powdery*, which suggests that this direction (solid line in Figure 3 left) can be regarded as the fresh dimension. Scores of freshness (S_freshness_) were obtained as the distance between the orthogonal projection of odor classes (dots in the figure) over the solid line and the right end of this line, measured in a metric arbitrary scale. Most of these odor classes can be directly matched with attributes in the B-H database. *Herbaceous* and *pyrogeneous* were paired with *vegetable* and *smoky*, respectively, based on their odor similarity. If S_freshness_ is compared with p_1_ loadings from the B-H database, it turns out that the correlation is statistically significant (*r* = 0.84, *p* = 0.0003), which indicates that freshness is an underlying dimension of this odor map. Interestingly, the orthogonal direction to the solid line in Figure 3 is defined by *floral vs. earthy*, which are also located at opposite positions in Figure 1. However, the position of *fruity* is arguable taking into account that this descriptor is usually regarded as fresh [3].

**Figure 4.**
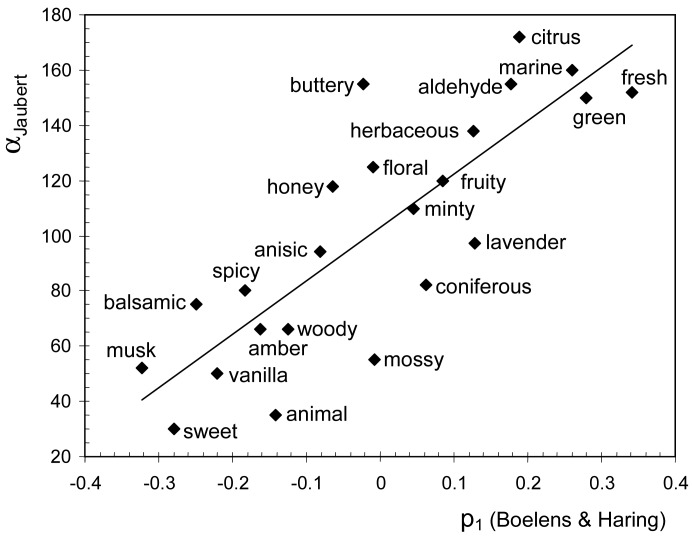
Plot of fitted regression analysis of α_Jaubert_ (angular coordinate of odor classes in the semicircular Field of Odors [15], measured in degrees) *vs.* p_1_ loadings of equivalent descriptors in the B-H database.

The Field of Odors [15] displays different odor classes from *citrus* in one end to *animal* at the other, which suggests that categories are arranged according to perceptual freshness. In order to study this issue, I compared the angular position (α) of each class with the p_1_ loading of equivalent attributes in the B-H database. The descriptors *vegetable*, *earthy*, *powdery*, *erogenic*, *aromatic*, *coniferous*, *watery*, and *sweet* from the B-H database were paired with *herbaceous*, *mossy*, *musk*, *amber*, *vanilla*, *terpenic*, *marine* and *caramel*, respectively, from the Field of Odors, which refer to similar smells. The correlation between α and p_1_ is statistically significant (*r* = 0.82, *p* < 10^−4^), which confirms that freshness is also the most salient dimension in the Field of Odors. Curiously, a similar correlation coefficient is obtained in Figure 2 (*r* = 0.86) and Figure 3 (*r* = 0.84). It is important to keep in mind that odor descriptors are not always interpreted in the same way, which would partly explain the residual variability observed in Figures 2–4. For example, eugenol is usually regarded as the reference material for *spicy*, but some perfumers may choose cinnamon [20], which smells sweeter.

### 3.2. Relationship between Fresh Odor Character and Substantivity

Most reference materials and attributes in the B-H database have a direct correspondence with descriptors used by the H&R guide (Table 1). Some attributes were paired taking into account their similarity in perfumery: *erogenic—sensual; animal*—*castoreum*[23]; *earthy*—*mossy*[21]; *smoky*—*leathery*[14,21,24]; *powdery*—*warm*[2]. The p_1_ loadings and substantivity index (SI) of these descriptors are shown in Table 1. Table 2 displays all reference materials in the B-H database (except *buttery*) and indicates p_1_, AI, SI, S_GS_, and evaporation index (EI). Strikingly, p_1_ is tightly correlated with SI (Table 1: *r* = −0.81, *p* < 10^−4^) and with EI^0.5^ (Table 2: *r* = 0.70, *p* = 0.005). The square root transformation (*i.e.*, EI^0.5^) is required to normalize the data. Moreover, AI is also significantly correlated with p_1_ (*r* = 0.75, *p* < 10^−4^), SI (*r* = −0.63, *p* = 0.001), S_GS_ (*r* = −0.68, *p* = 0.0003) and with EI^0.5^ (*r* = 0.72, *p* = 0.006). Taking into account that (i) antierogenic and refreshing are equivalent concepts [4], and (ii) p_1_ loadings can be interpreted as sensory ratings on a scale of freshness, the observed correlation among p_1_, AI, SI, S_GS_, and EI reveals that materials with a low substantivity tend to be perceived as fresh or refreshing.

In Thiboud's database, certain material was described with a given attribute as main or ancillary descriptor if that odor character was clearly recognizable or just noticeable, respectively. Because of this, odor descriptors can be coded numerically as 0 if that term is not applied, 1 if it is applied as ancillary descriptor, and 2 for the main attributes. These coded descriptions are shown in Table 3 for 6 relevant attributes: (i) *fresh* and *citrus*, which account for the fresh odor character, (ii) *balsamic* and *oriental*, which are dissimilar to *fresh*, and (iii) *floral* and *agrestic*, with an intermediate refreshing tone (Figure 1). By adding the values in columns *fresh*, *citrus*, *floral* and *agrestic*, and subtracting the columns *balsamic* and *oriental*, it results a numeric variable from −3 to 6 that can be interpreted as sensory scores on a fresh scale (S_fresh_ in Table 3). Interestingly, S_fresh_ is significantly correlated with SI (*r* = −0.85, *p* < 10^−4^), CS (*r* = −0.77, *p* < 10^−4^) and S_GS_ (*r* = −0.61, *p* < 10^−4^). The tight correlation once again evidences that fresh odor character reflects odorant tenacity.

Substantivity and AI values of additional materials not included in Table 3 are shown in Table 4. If both tables are merged, it turns out that SI is strongly correlated with CS (*r* = 0.88, *p* < 10^−4^) and S_GS_ (*r* = 0.83, *p* < 10^−4^), but S_GS_ yields a weaker correlation with EI^0.5^ (*r* = −0.61, *p* = 0.0001) probably because many EI values are missing for the least volatile materials.

**Figure 5.**
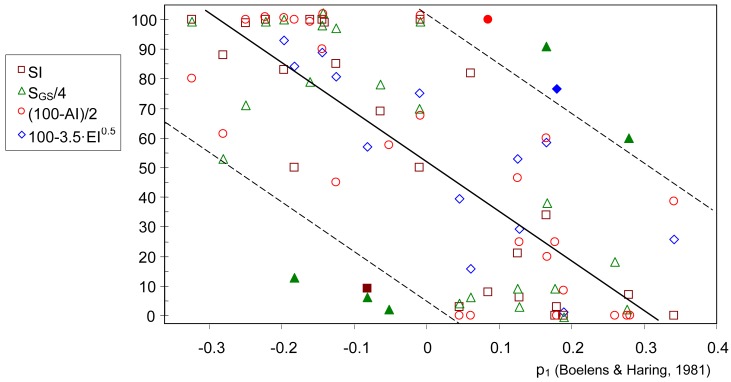
Relationship between fresh odor character and substantivity of reference materials used by Boelens and Haring [12]: scatterplot of p_1_ (loadings in the formation of PC1) *vs.* antierogenic index (AI) and substantivity parameters: SI, S_GS_ and EI (values in Table 2). AI, S_GS_ and EI were conveniently transformed as indicated in the legend to range approximately on a 0–100 scale. The fitted regression line (*r* = −0.81) was obtained after discarding 8 outliers (filled points, highlighted in bold in Table 2). Dashed lines: prediction limits with a confidence level of 95%.

Poucher [18] classified odorants as top notes (CS < 15), middle notes (15 ≤ CS ≤ 60) and base notes (CS >60). Interestingly, most materials listed in Table 3 up to elemi oil are basically encountered in top notes (SI ≪ 50) and their CS is below 15. Moreover, materials from vetiver oil to tolu can be regarded as base notes (SI ≈ 100) and they exhibit a high substantivity (CS ≫ 60). Thus, Poucher's classification based on CS values seems adequate. Some materials with a low CS (top notes) like mimosa absolute, narcissus absolute or copaiba present a high substantivity according to The Good Scents Company (S_GS_ = 400), which suggests that such particular values might not be reliable. Actually, the website does not indicate how this parameter was obtained. Moreover, 5 out of the 8 outliers highlighted in Figure 5 correspond to S_GS_ values.

Most materials in Table 2 with p_1_ > 0 smell antierogenic (AI > 0) and, as a result, the correlation between p_1_ and AI is statistically significant (*r* = 0.75, *p* < 10^−4^). In Table 3, AI is positively correlated with S_fresh_ (*r* = 0.42, *p* = 0.01). By merging Tables 3 and 4, it turns out that AI is also correlated with SI (*r* = −0.60, *p* < 10^−4^), S_GS_ (*r* = −0.50, *p* = 0.0004) and CS (*r* = −0.45, *p* = 0.0006). Given the relationship between substantivity and freshness, the observed correlations support Jellinek's interpretation of *antierogenous* and *refreshing* as equivalent concepts in odor description. Nonetheless, most floral odors were regarded by Jellinek [4] as erogenic (AI ≤ −50), which is somewhat unexpected because floral scents present an intermediate refreshing tone (Figure 1) and substantivity (SI ≈ 50 in Table 4). The reason might be that the three perfumers who obtained AI values were probably men. Floral scents, which are perceived as feminine, may be regarded by men as erogenic (*i.e.*, arousing sexual desire for the opposite sex). Thus, a larger panel should have been used with the same number of male and female individuals. Actually, Jellinek [4] recognized that his results were preliminary because of the difficulties in reaching an agreement among observers.

## 4. Discussion

### 4.1. Freshness as a Salient Dimension of Odor Perception

Smell is a complex multidimensional perception difficult to measure and describe [26]. The underlying constructs of this multivariate space are still poorly understood. A relatively easy procedure is proposed here to study if freshness is a salient dimension of odor maps, by assigning a refreshing tone to odor character descriptors. This issue is of interest for developing standard sensory maps of cosmetic odors.

Different works have reported that freshness is a latent dimension in the perceptual space of fragrances (for review, see [2]). However, this idea is not well established yet in olfactory research. Actually, neither *fresh* nor *refreshing* are included in the comprehensive Dravnieks' list of 146 terms most commonly used in odor description [27]. Moreover, the fresh dimension was not considered by some studies that have discussed several odor maps of perfumery scents [13,28]. Edwards classifies 12% of fragrances in the citrus, green, watery or fruity categories, which are grouped in the fresh family [2]. But *fresh* is not considered by most fragrance companies as a main olfactory family of perfumes (see Table 1 of [29]). This category is also missing in the classification of fragrances proposed by the French Society of Perfumers [30], Fragrantica, or Osmoz by Firmenich, among many others. The reason could be the lack of consensus in the interpretation of *fresh* in odor description, as discussed below.

Many psychophysical studies have reported that pleasantness is the most salient dimension when a wide range of odors are assessed (for review, see [31]). This dimension did not clearly show up in a previous study of the B-H database by checking the correlation between loadings and hedonic tones from the literature [11]. Although the same methodology could have been applied here, there is enough evidence to support that freshness is the basic construct underlying Figures 2–4. Actually, *animal*, *mossy* or *earthy* are often described as disagreeable odors, but these descriptors appear close to *sweet* or *spicy* that are regarded as pleasant.

### 4.2. Proposed Hypothesis to Explain the High Substantivity of Erogenic Odors

According to Jellinek [4], erogenous scents are those arousing sexual desire. They are basically reminiscent of the human body smell (*i.e.*, odors released by our skin) and somewhat resemble the smell of materials obtained from animals (e.g., civet, castoreum, ambergris or Tonkin musk). This similarity between *erogenic* and *animal* is apparent in the B-H database because both descriptors are correlated (*r* = 0.46) and determine an independent dimension of odor character (see Figure 3 of [2]). Body odorants are obviously easily retained by the skin and, hence, they present a high substantivity. The same applies to animalic materials. This reasoning is consistent with the observed negative correlation between antierogenic odor character and substantivity parameters (Tables 2–4).

Taking into account that substantivity depends on physicochemical properties, the tight correlation of substantivity with freshness implies that, in the case of aroma chemicals, their refreshing odor character could be estimated by means of electronic noses [32] and also based on physical properties. In the B-H database, the projections of odorants over PC1 can be interpreted as estimated scores of freshness on a numeric scale. Work in progress suggests that these scores can be predicted from a set of 20 physicochemical parameters and molecular descriptors using multiple linear regression, with a coefficient of determination R^2^ ≈ 0.52.

This preliminary result further suggests that the correlation of fresh odor character with substantivity is primarily innate and evolutionarily hardwired. Nonetheless, olfactory perception of fragrance raw materials can drastically change when applied in a mixture (e.g., a perfume), in a paper blotter or on the skin, thus possibly changing its substantivity. Further research will be necessary to study this issue. The development of standard psychophysical procedures to obtain accurate values of substantivity and scores of freshness for perfumery materials is strongly encouraged.

### 4.3. Conditioned and Unconditioned Factors Influencing Odor Quality Perception

It is well established that odor quality perception is strongly influenced by memory [33], learning [34] and culture [35,36]. Social effects affecting the perception of fragrances have also been discussed [37]. The human fetus starts the learning process of smelling in the prenatal environment [38]. Flavors from the mother's diet during pregnancy are transmitted to amniotic fluid and swallowed by the fetus [39], which may provide the foundation for cultural and ethnic differences in odor preference.

Despite the general belief that odor perception is basically shaped by learned associations, some studies reveal that certain aspects of olfactory perception might be innate and hardwired. For example, rats show an unconditioned defensive response to the odor of predators [40]. Moreover, recent works have revealed that the hedonic odor character is partly determined by molecular structure [31]. Interestingly, freshness is the most salient perceptual dimension of cosmetic scents and it is also somewhat encoded by molecular structure given the correlation with substantivity. Although many efforts have been carried out to relate molecular structure to the perceived smell [41], odor-structure relationships focused on the fresh olfactory quality have not been reported yet as far as I know. Ferdenzi *et al.*[42] found that the refreshing/energizing dimension of smell was common to three tested cultures, but other constructs were different. The neurological basis of innate and learned responses to odors has been investigated in mice [43].

### 4.4. Interpretation of ‘Fresh’ vs. ‘Refreshing’ in Odor Description

The *Collins Dictionary & Thesaurus*[44] considers the following terms as synonyms of *refreshing*: new, original, novel, unusual, stimulating, innovative, cooling, invigorating, *etc.*. Probably based on the semantic associations, different studies have found that *refreshing* and *invigorating* are similar odor descriptors [19]. Actually, a fresh fragrance is usually considered invigorating, nature inspired, reminiscent of early morning air or sea breeze and it is typified by green, citrus notes [1]. Chrea *et al.*[45] found that *refreshed* and *revitalized* were interpreted in the same way by French speakers, and both terms were classified in an emotional factor of odor perception called energizing/refreshing. The same association was found in a further study carried out in Liverpool (UK) and Singapore, and the underlying factor was named *energy*[42]. *Refreshing* and *stimulating* are regarded as orthogonal dimensions of the Odor Effects Diagram [4], but this issue is arguable. In the odor map proposed by Tisserand [46], *vivacity* and *stimulant* appear next to *erogenic*, which is also debatable based on the reported empirical evidence.

Given the multiple meanings of *refreshing*, this term might be interpreted differently according to culture, language, context, age, and experience. This issue may explain why Jellinek [4] regarded *refreshing* and *fresh* as different odor descriptors. The former was associated with antierogenous and the latter with green–herbaceous odors, which were supposed to produce antierogenous and stimulating effects. However, this criterion is arguable given the semantic association between *refreshing* and *stimulating*. Moreover, odors considered by Jellinek [4] as stimulating are better described as masculine [2]. Although *fresh* and *refreshing* may have different connotations in odor description according to language, their different meaning is not clearly established in perfumery and, hence, in my opinion both terms can be used as synonyms for clarity purposes.

### 4.5. Controversies in the Interpretation of ‘Fresh’ in Odor Description

The association between *refreshing* and *energizing* might correspond to the activation dimension of emotion (arousal) [45], but another interpretation is possible. According to Jellinek [29], the refreshing dimension underlies consumers' perceptions as to whether a fragrance seems to be more suited for formal evening wear or for informal daytime wear. People feel more comfortable wearing during the day fragrances that do not transmit an erotic message (*i.e.*, smelling antierogenic), which seems quite obvious. Thus, refreshing scents are preferred for daytime wear because they are not perceived as erotic, and daytime is associated with stimulating activities, energy, vigorous actions, *etc*.

Although *fresh* was long ago proposed as an independent category of odors [47], this particular odor quality is rather subjective and it can be interpreted differently. According to Müller [24], *fresh* is generally associated in European regions with lemon, lavender, green notes and light floral components. Different studies support this interpretation [2]. For example, a so-called *light* dimension determined by the descriptors *fresh* and *lemon* was found by analyzing numeric odor profiles of 40 compounds rated by French individuals [48]. However, Müller states that sweet and powdery perfumes are also considered as fresh in North America [24]. Taking into account that *sweet* and *powdery* are perceived as dissimilar to *fresh*[2], it is of interest to discuss this controversy.

*Fresh* can be defined in English as (i) newly made or obtained, novel, original, additional, (ii) not stale or deteriorated, (iii) not canned, frozen or preserved, *etc.*[49]. Consistent with this definition, Schiffman *et al.*[50] regarded *fresh* and *rotten* as two polarities of the same semantic differential scale. By contrast, the first meaning of *fresh* in French (frais) and Spanish (fresco) is ‘moderately cool’ [51,52], which is antonym of *warm*. The semantic association between *fresh* and *cool* also exists in English, but only when applied to weather, wind or breeze. Thus, fresh air means clean and cool, found outside buildings rather than in a room [49]. In French, the similarity between *refreshed*, *clean* and *cooling* in odor perception has also been reported [45].

I believe that *fresh* was used originally in the sense of *cool*, probably long ago by French perfumers. Unfortunately, *fresh* and *cool* cannot be regarded nowadays as synonyms because *cool* has acquired many connotations in perfumery [1]. *Cool* is often applied to the perception of freshness associated with a trigeminal effect, as it is the case of minty scents. Actually, *minty* yields the highest correlation with ‘cool, cooling’ (*r* = 0.82) in the database of Dravnieks [27]. Moreover, Harper [53] selected two odorants sharing a minty–fresh odor character (menthol and camphor) as references for ‘cool, cooling’. In order to avoid confusion, I would recommend using *fresh*, *refreshing* and *antierogenic* as synonyms, referred to scents perceived as cooling and not associated with a trigeminal effect.

### 4.6. Fresh and Warm as Opposite Polarities of a Perceptual Odor Dimension

Based on the semantic meaning, French and Spanish people would easily agree that *fresh* and *warm* are two opposite poles of the same perceptual odor dimension, because they will readily associate *fresh* with *cool*. The same link probably exists in Japanese, because a *fresh* dimension that discriminated *cold* and *sour* with respect to *warm*, *sweet* and *sexy* was found by Japanese researchers who analyzed with PCA numeric odor profiles of 37 aroma chemicals according to 55 descriptors [32]. Conversely, in English, not everybody would understand the dissimilarity *fresh vs. warm* unless it is indicated that *fresh* is used as a synonym of *cool*. The lack of consensus in the semantic interpretation of freshness might explain why this perception is not easy to define in perfumery [1,24].

*Refreshing* and *erogenous* were regarded by Jellinek [4] as opposite polarities of the same dimension. Consistent with this criterion, AI values are positively correlated with p_1_ (Table 2) and S_fresh_ (Table 3). In the B-H database, the negative correlation between *fresh* and *erogenic* (*r* = −0.24, *p* < 10^−4^) partly supports Jellineks' interpretation, but *fresh* yields the most negative correlation with *powdery* (*r* = −0.58). The reference material for this descriptor smells warm [2], which suggests that the perceptual dimension of freshness is better interpreted as a fresh *vs.* warm polarity rather than fresh *vs.* erogenous.

This interpretation is supported by further evidence from the literature. Warm perfumes have a high proportion of animalic ingredients [24] and are often described as rich and tenacious [1]. Harper [53] selected costus and amber as standards for *warm*. Both materials were regarded by Boelens & Haring [12] as references for *erogenic*, which implies that *warm*, *erogenic* and *animal* are similar descriptors in perfumery. Actually, in a two-dimensional odor map recently reported, *warm* and *sexy* appeared close to each other [45]. The origin of this similarity from an evolutionary perspective is not well understood yet, but some hypotheses have been proposed [54].

In a reported analysis of the language of French perfume advertising, it was found that the dimension warm *vs.* fresh was the most important axis of the semantic field of fragrances [55]. Chrea *et al.*[45] obtained a two-dimensional odor map from numeric ratings of 24 odorant samples according to 73 affective terms in French, and it was found that *refreshed* and *warm* appeared in opposite positions within the pleasant cluster of terms. Jellinek [29] developed a two-dimensional map of fragrances and one of the dimensions was interpreted as warm *vs.* cool. In a previous study, 140 commercial perfumes were also classified according to a cool *vs.* warm dimension [56]. Given that *cool* and *warm* are terms referring to temperature, these studies support the interpretation of *fresh* and *warm* as opposite polarities of a perceptual odor dimension somehow associated with temperature. Interestingly, colors can also be described on a warm *vs.* cool scale [57].

### 4.7. Proposed Hypotheses to Explain the Fresh/Warm Dimension of Odor Character

Taking into account the alternation of seasons in temperate climates, with cool temperatures in winter and warmer conditions in summer, the ability of human olfaction to perceive scents as fresh *vs.* warm might be explained from an evolutionary perspective. In summer, as a result of the hot weather, only those odorants with higher substantivity would be found in the natural environment, while light scents could be encountered at cooler temperatures. This hypothesis is consistent with the fact that fresh scents are characterized by a lower substantivity, while the opposite applies to warm odors.

The idea that olfactory perceptual space in humans might reflect chronobiological annual rhythms was put forward by Tisserand [46]. Starting from the four elements (air, fire, earth and water) long ago proposed by Greek philosophers like Hippocrates, he discussed their associations with the four seasons and with moods evoked by essential oils. It was found that the resulting structure had certain resemblance to the Odor Effects Diagram [4]. This diagram has been recently discussed in detail [2]. Tisserand suggested that erogenic scents evoked odors typically encountered in summer, while those associated with winter such as *watery* were regarded as antierogenic. Autumn was associated with *earthy*, which is intuitively appealing because this descriptor has a neutral refreshing tone (p_1_ ≈ 0). Moreover, *earthy* is the attribute most dissimilar to *floral* (Figure 1), and the latter would correspond to the typical smells of spring.

The olfactory map shown in Figure 1 might reflect an underlying structure of odor perception that discriminates those scents most typically encountered in the different seasons in temperate climates: *fresh* (winter), *floral* (spring), *warm* (summer), and *earthy* (autumn). The position of *summer* next to *fruity* in Figure 1 might suggest that summer scents are perceived as fresh, which is misleading. The correct interpretation is that fresh scents are preferred in summer, probably because they evoke cool temperatures. Conversely, warm and animalic fragrances are preferred in winter [29].

The work of Tisserand [46] leads to the hypothesis that human olfactory perception was shaped throughout evolution in temperate climates to recognize the characteristic odors of each season. This theory is debatable because our sense of smell did not evolve under a constant climate. Moreover, it is uncertain if the fresh odor character is perceived differently by human races that developed in climates with slight weather variations along the year, or by people living in such climates. The proposed theory seems very simplistic given the complexity of factors affecting olfactory perception, but it deserves to be further investigated because it might provide the fundamental basis to better understand certain psychological aspects of olfactory perception. For example, it is still poorly understood to what extent the perception of freshness depends on culture, language, context, age, *etc*. It would also be of interest to study why sweet fragrances are preferred by women, or why cosmetic odors are perceived as masculine *vs.* feminine.

## 5. Conclusions

Odor freshness is an underlying dimension in the perceptual space of perfumery scents, as further studied here. Fresh odors are typical in top notes, while those most dissimilar to *fresh* are encountered in base notes. This relationship between freshness and substantivity is well known by perfumers, but it has not received much attention yet by the scientific community. The present work reports that the correlation between perceptual freshness and odorant substantivity is quite strong (p_1_*vs.* SI in Table 1: *r* = −0.82; S_fresh_*vs.* SI in Table 3: *r* = −0.85). This result is important because substantivity depends on physicochemical properties, which provides the fundamental basis to predict odor freshness based on molecular structure.

The interpretation of *fresh* in perfumery as synonym of *cool* and antonym of *warm* suggests that this psychological dimension might be associated with temperature and may reflect a structure of odor perception shaped throughout evolution in temperate climates. Thus, *fresh* would correspond to scents typically encountered in the cool season, while *warm* would be evoked by odors found in nature during summer.

## Figures and Tables

**Table 1.** Correspondence between attributes/reference materials in the B-H database and descriptors in the H&R Fragrance Guide [17]. A substantivity index (SI) is calculated according to Equation (1) based on the frequency of occurrence of odor descriptors used to describe the top (N_T_), middle (N_M_) and base note (N_B_) of 820 commercial perfumes.

Attribute a	p_1_	**H&R Fragrance Guide**					Attribute a	p_1_	**H&R Fragrance Guide**				
	
		Descriptor	N_T_	N_M_	N_B_	SI			Descriptor	N_T_	N_M_	N_B_	SI
*bergamot oil*	0.341	bergamot	722	0	0	**0**	floral	−0.010	floral	86	681	47	**48**
fresh	0.341	fresh	576	63	8	**6**	honey	−0.064	honey	3	50	38	**69**
green	0.279	green	240	35	1	**7**	anisic	−0.081	anise	50	11	0	**9**
watery	0.260	watery	0	1	0		*cedarwood oil*	−0.124	cedarwood	0	159	364	**85**
*lemon oil*	0.189	lemon	401	1	0	**0**	woody	−0.124	woody	3	166	305	**82**
citrusy	0.189	citrusy	57	0	0	**0**	*civet absolute*	−0.142	civet	0	0	199	**100**
*galbanum res.*	0.180	galbanum	113	7	0	**3**	animal	−0.142	castoreum	0	0	83	**100**
tart (dry)	0.180	dry	19	63	0	**38**	smoky	−0.144	leathery	0	1	104	**100**
aldehyde	0.177	aldehydic	140	0	0	**0**	*amber.+costus^e^*	−0.161	ambery	0	0	137	**100**
*bay oil*	0.165	bay	5	11	0	**34**	erogenic	−0.161	sensual	0	0	70	**100**
*lavender oil*	0.128	lavender	204	26	0	**6**	*eugenol*	−0.182	clove f	0	26	0	**50**
*clary sage oil*	0.126	clary sage	83	62	0	**21**	spicy	−0.182	spicy	163	244	1	**30**
vegetable	0.126	herbaceous	165	7	2	**3**	*patchouli oil*	−0.197	patchouli	0	136	253	**83**
fruity	0.085	fruity	151	24	3	**8**	*vanillin*	−0.221	vanilla	0	0	301	**100**
fruity	0.085	peach b	149	7	0	**2**	*olibanum res.*	−0.249	olibanum	0	3	117	**99**
*fir needle oil*	0.061	fir c	0	24	41	**82**	balsamic	−0.249	balsamic	0	0	76	**100**
coniferous	0.061	pine c	1	87	0	**49**	sweet	−0.280	sweet	0	50	157	**88**
*peppermint oil*	0.045	peppermint d	29	2	0	**3**	sweet	−0.280	tonka g	0	1	287	**100**
*oakmoss res.*	−0.008	oakmoss	0	0	139	**100**	*musk+coumarin* h	−0.323	musk	0	0	698	**100**
earthy	−0.008	mossy	0	0	252	**100**	powdery	−0.323	powdery	0	0	376	**100**
*jasmine absolute*	−0.010	jasmine	0	671	0	**50**	powdery	−0.323	warm	0	0	137	**100**

a Reference materials (in *italics*) and descriptors are listed by decreasing order of p_1_ (loadings in the formation of the first principal component). The term ‘res.’ stands for resinoid. The correspondence between descriptors and references used by Boelens & Haring [12] is indicated in Table 2.

b Peach is the fruity descriptor most frequently encountered in the H&R guide.

c Pine and fir trees are conifers and their essential oil smells alike.

d Peppermint or spearmint.

e Mixture of ambergris and costus oil.

f Clove oil contains >85% of eugenol [25].

g Tonka is the second attribute (after vanilla) most frequently associated to *sweet* in the H&R guide.

h Mixture of musk ketone and coumarin.

**Table 2.** Substantivity (SI: substantivity index from Table 1; S_GS_: substantivity value from www.thegoodscentscompany.com; EI: evaporation index [7]) and fresh odor character (p_1_: loadings in the formation of PC1; AI: antierogenic index [4]) of reference materials used by Boelens & Haring [12]. Values in bold appear as outliers in Figure 5.

Reference material	Attribute	**Freshness**		**Substantivity**		
	
		p_1_	AI	SI	S_GS_	EI
Bergamot oil	Fresh	0.341	23	0		450
Methyl 2-octynoate	Green	0.279	100	7	**240**	
Styrallyl acetate	Sourish	0.277	100		8	
Cyclamen aldehyde	Watery	0.260	100		72	
Lemon oil	Citrusy	0.189	83	0	4	800 d
Galbanum resinoid	Tart (dry)	0.180	100	3		**45**
Aldehyde C-10	Aldehyde	0.177	50	0	36	
10-undecen-1-ol	Fatty	0.166	60		152	
Bay oil	Metallic	0.165	−20	34	364	140
Lavender oil	Lavender	0.128	50	6	12	410
Clary sage oil	Vegetable	0.126	7	21	36	180
Hexadecanal	Fruity	0.085	**−100**	8		
Fir needle oil	Coniferous	0.061	100	82	24	580
Peppermint oil	Minty	0.045	100 a	3	16	300
Oakmoss resinoid	Earthy	−0.008	−35	100	400	
Jasmine absolute	Floral	−0.010	−100	50	280	50
Methyl salicylate	Medicinal	−0.052	−15		**8**	
Ethyl phenylacetate	Honey	−0.064		69	312	
Fennel oil	Anisic	−0.081		**9**	**24**	150
Cedarwood oil	Woody	−0.124	10	85	388	30
Civet absolute	Animal	−0.142	−100	100	400	
Cade oil	Smoky	−0.144	−80 b	100	400	10
Ambergris + costus oil	Erogenic	−0.161	−100	100	316^c^	
Eugenol	Spicy	−0.182	−100	50	**52**	20 e
Patchouli oil	Dusty	−0.197	−100	83	400	4
Vanillin	Aromatic	−0.221	−100	100	400	
Olibanum resinoid	Balsamic	−0.249	−100	99	284	
Heliotropin	Sweet	−0.280	−23	88	212	
Musk ketone+coumarin	Powdery	−0.323	−60	100	400	

a Value of spearmint (both plants are botanically related).

b Value of birch tar oil (both smell smoky and are obtained by destructive distillation of wood).

c Value of costus oil (S_GS_ of ambergris is not available).

d Value of lime oil.

e Value of clove oil.

**Table 3.** Substantivity parameters and fresh odor character of the 44 natural materials contained in the semantic odor profile database compiled by Thiboud [1].

Material a	**Substantivity**				**Odor Character Descriptors** f						**Freshness**	
		
	CS b	SI c	S_GS_^d^	EI e	I_fresh_	I_citrus_	I_agrest_	I_floral_	I_balsam_	I_oriental_	S_fresh_^g^	AI h
Petitgrain bergamot oil	3	4	28	170	2	2	1	1	0	0	6	100
Bergamot oil	6	0		450	2	2	1	0	0	0	5	23
Jonquil absolute	24				2	0	1	2	0	0	5	−100
Lime oil	2	0	20	800	2	2	0	0	0	0	4	80
Mandarin oil	2	4	8		2	2	0	0	0	0	4	−27
Coriander oil	3	13	8	200	2	0	2	0	0	0	4	−5
Grapefruit oil	6		264		2	2	0	0	0	0	4	
Thyme oil	7	39	172	220	2	0	2	0	0	0	4	100
Lemon oil	8	0	4		2	2	0	0	0	0	4	83
Orange Florida oil	11	1	140	970	2	2	0	0	0	0	4	10
Mimosa absolute	14		400		0	0	2	2	0	0	4	−30
Mugwort oil	9	4	16		1	0	2	0	0	0	3	
Chamomile oil, Roman	10		112	530	1	0	2	0	0	0	3	−100
Wormwood oil (absinthe)	10		212	500	0	0	2	1	0	0	3	
Violet leaf absolute	18	23	400	20	1	0	0	2	0	0	3	0
Cumin oil	4	7			0	0	2	0	0	0	2	−40
Myrtle oil	4			400	0	0	2	2	2	0	2	
Rose oil (Bulgarian)	8	50	168	10	1	0	0	1	0	0	2	−80
Galbanum oil	11	3	72	540	1	0	1	0	0	0	2	100
Carrot seed oil	11		96	80	0	0	2	0	0	0	2	−60
Elemi oil	13		20	720	2	1	1	0	2	0	2	100
								
Clary sage oil	20	21	36	180	0	0	2	0	0	0	2	7
Geranium Bourbon oil	29	50	28	120	1	0	0	1	0	0	2	−70
Tuberose absolute	43	50	304		0	0	0	2	0	0	2	−100
Cardamom oil	30	16		320	0	0	2	0	0	1	1	75
Rose absolute (French)	43	50	168		0	0	0	1	0	0	1	−90
Cedarwood oil Virginia	8	85	388	30	0	0	1	0	0	0	1	10
Narcissus absolute	11	49	400		0	0	0	2	1	0	1	
Nutmeg oil	11		52	550	0	0	1	0	0	1	0	−40
Ginger	7		292	150	0	0	0	0	0	0	0	0
Copaiba	6		400		0	0	0	2	2	0	0	
Clove bud oil	22	50	188	20	0	0	0	1	0	1	0	−100
Cinnamon leaf oil	22	56	304	30	0	0	1	0	0	1	0	
								
Vetiver oil Bourbon	100	86	400		0	0	0	0	0	0	0	100
Patchouli oil	100	83	400	4	0	0	1	0	0	1	0	−100
Cistus oil (labdanum)	100	100	400		0	0	2	0	2	0	0	−80
Styrax	100	98	400		0	0	0	2	2	0	0	−100
Pepper (black) oil	100		48	400	0	0	0	0	0	1	−1	
Opoponax	90	100	400	20	0	1	0	0	2	1	−2	10
Allspice (pimento berry)	100		400	50	0	0	0	0	2	0	−2	−40
Sandalwood oil	100	88	400		0	0	0	0	2	1	−3	−63
Peru	100		400		0	0	0	0	2	1	−3	−100
Benjoin	100	100	400		0	0	0	0	2	1	−3	−100
Tolu	100	100	400		0	0	0	0	2	1	−3	−100

a Sorted by decreasing value of S_fresh_.

b Coefficient of substantivity [18].

c Substantivity index deduced from the H&R Fragrance Guide according to Equation (1).

d Substantivity (hours) according to www.thegoodscentscompany.com.

e Evaporation index [7].

f Indicator variables corresponding to 6 relevant descriptors. The value 2 indicates a main descriptor in the semantic odor profile, and the value 1 indicates an ancillary descriptor.

g Scores of freshness calculated as: I_fresh_ + I_citrus_ + I_agrestic_ + I_floral_ − I_balsamic_ − I_oriental_.

h Antierogenic index [4].

**Table 4.** Substantivity parameters and antierogenic index (AI) of materials not included in Table 3 (sorted approximately by increasing order of substantivity).

Material	**Substantivity** a				AI	Material	**Substantivity** a				AI
	
	CS	SI	S_GS_	EI			CS	SI	S_GS_	EI	
Lavender oil	4	6	12	410	50	Cascarilla oil	29		248	200	0
Neroli oil	5	4	116	210	17	Orange flower absolute	31	15	400		−50
Laurel leaf oil	9			750		Cinnamon bark oil	24	56	372	120	−100
Peppermint oil	8	3	16	300	100	Lily of the valley; orchid		50			
Rosewood oil	2	16	12	250	−8	Carnation absolute		50			−30
Hyacinth absolute	11	16			40	Ylang-ylang oil	32	50	140	90	−80
Basil oil	14	6		225	−50	Ambrette seed oil	30		121	20	−100
Palmarosa oil	14		60	50	0	Orris resinoid	40 b	50			−60
Juniper berry oil		18		700		Jasmine absolute	43	50	280	50	−100
Gardenia		14				Heliotrope		70			
Rosemary oil	21	5	4	820	100	Civet absolute	79	100	400		−100
Marjoram oil	18		12	600		Tonka resinoid	100	100	400		−55
Verbena resinoid	19				0	Castoreum absolute	100	100	400		−100

a CS: coefficient of subst.; SI: subst. index; S_GS_: subst. from www.thegoodscentscompany.com; EI: evaporation index.

b Average CS of orris concrete, orris absolute and orris oleo-resin.
